# Enhanced Cognitive Inhibition in Table Tennis Athletes: Insights from Color-Word and Spatial Stroop Tasks

**DOI:** 10.3390/brainsci14050443

**Published:** 2024-04-29

**Authors:** Qin Huang, Xuechen Mao, Jilong Shi, Jun Pan, Anmin Li

**Affiliations:** 1 School of Psychology, Shanghai University of Sport, Shanghai 200438, China; 2011916004@sus.edu.cn (Q.H.); xuechenmao.asprin@outlook.com (X.M.); shijilong096@163.com (J.S.); yanjunp@gmail.com (J.P.); 2 Center for Exercise and Brain Science, Shanghai University of Sport, Shanghai 200438, China

**Keywords:** conflict control, cognitive inhibition, Stroop effect, table tennis athletes, ERP

## Abstract

The ability to inhibit conflicting information is pivotal in the dynamic and high-speed context of fast-ball sports. However, the behavioral and electrophysiological characteristics underlying the cognitive inhibition processes associated with table tennis expertise remain unexplored. This study aims to bridge these research gaps by utilizing the color-word Stroop task and the spatial Stroop task alongside event-related potential (ERP) measurements to investigate domain-general and domain-specific cognitive inhibition among table tennis athletes. The study involved a total of 40 participants, including 20 table tennis athletes (11 males and 9 females; mean age 20.75 years) and 20 nonathletes (9 males and 11 females; mean age 19.80 years). The group differences in the Stroop effect on behavioral outcomes and ERP amplitudes were compared within each task, respectively. In the color-word Stroop tasks, athletes exhibited smaller incongruent-related negative potential amplitudes (N_inc_; 300–400 ms; *p* = 0.036) and a diminished Stroop effect on late sustained potential amplitudes (LSP; 500–650 ms; *p* = 0.028) than nonathletes, although no significant differences were observed in behavioral outcomes (*p* > 0.05). Conversely, in the spatial Stroop tasks, athletes not only responded more swiftly but also exhibited reduced Stroop effects on both LSP amplitudes (350–500 ms; *p* = 0.004) and reaction times (*p* = 0.002) relative to nonathletes. These findings suggest that table tennis athletes excel in cognitive inhibition in the context of both domain-general and domain-specific tasks, particularly exhibiting enhanced performance in tasks that are closely aligned with the demands of their sport. Our results support the neural efficiency hypothesis and improve our understanding of the interactions between cognitive functions and table tennis expertise.

## 1. Introduction

Sports-cognitive interactions have been widely debated in recent decades. On the one hand, the cognitive system plays a crucial role in the planning, preparation, and control of complex motor processes [1], which are positively linked to athletes’ performance [2,3]. On the other hand, athletes exhibit cognitive advantages over nonathletes [4,5,6]. The multifactorial gene‒environment interaction (MGI) model posits that expert advantages could result from deliberate practices that impact brain mechanisms [7]. Studies have shown that extended training can lead to adaptive changes in the cognitive functions of the nervous system [8,9]. However, as the scope of research has broadened, it has become evident that athletes across different sports exhibit unique cognitive skills [10,11,12,13]. This situation has led researchers to call for the investigation of cognitive functions specific to individual sports [14]. Recent studies have focused on how expertise in table tennis affects conflict control, an advanced cognitive function.

Conflict control is essential with regard to managing the intrusion of irrelevant information in the context of task performance and encompasses both response and cognitive conflict inhibition [15]. These forms of inhibition play critical roles in fast-paced sports such as table tennis, where rapid changes and quick decisions are critical [16,17]. Table tennis, which has been recognized worldwide as one of the quickest racket sports [18], requires athletes to predict their opponents’ intentions, identify relevant signals, and respond accurately within very short time frames. This process requires players not only to suppress inappropriate action impulses but also to inhibit the cognitive interference caused by opponents’ deceptive maneuvers [19]. Research has consistently shown that table tennis athletes exhibit enhanced response inhibition capabilities. Notably, elite table tennis players have been observed to exhibit quicker reaction times (RT) and greater accuracy (ACC) than nonathletes in the context of go/no-go tasks [20,21]. Moreover, table tennis players, compared to novices, exhibit decreased prefrontal cortex activity during tasks that require response inhibition [22]. These outcomes lend support to the neural efficiency hypothesis, which posits that expert individuals exhibit more effective neural activity [23,24].

Cognitive inhibition is usually determined using the Stroop paradigm. In the classical Stroop task [25], participants are instructed to name the color in which a color word is written (e.g., the word ‘RED’ printed in blue). The Stroop effect is indicative of the cognitive process required to overcome the interference caused by the irrelevant word meaning, thus shedding light on the mechanisms underlying cognitive inhibition [26,27]. Studies that have employed event-related potential (ERP) techniques have revealed the neural foundations of the conflict control processes associated with the Stroop effect. Notably, the frontal N2 and N4 components, which are associated with conflict detection, are believed to originate from the anterior cingulate cortex, which is associated with the brain’s response to cognitive conflict (for a review, see [28]). Incongruent stimuli typically generate larger negative amplitudes in the context of conflict monitoring, which is indicative of increased cognitive effort aimed at managing conflict and referred to collectively as the incongruent-related negative potential (N_inc_; [29]). This detection of conflict prompts the prefrontal cortex to exert top-down control to resolve the conflict, as evidenced by changes in the parietal late sustained potential (LSP; [30,31]).

According to the MGI model, the interplay between cognitive functions and systematic practice significantly improves the performance of experts [7]. Previous research has shown that cognitive inhibition, as measured by the Stroop task, benefits athletes in various sports, including road cycling [32], soccer [33,34], and both self-paced and externally paced sports [12]. Research on the cognitive inhibition capabilities of table tennis athletes, however, remains limited. Studies that have utilized Golden’s Stroop tests have demonstrated that both healthy [35] and para [36] elite table tennis athletes exhibit greater accuracy than controls in the context of reading the colors of Stroop words aloud. However, the “reading” task in the context of Golden’s Stroop tests requires minimal perceptual-motor processing under time pressure, unlike the demands associated with table tennis. Consequently, this situation highlights the need to investigate how table tennis expertise influences general cognitive inhibition ability by using laboratory-based color-word Stroop tasks that can simulate perceptual-motor and time pressures that resemble those associated with competitive sports more effectively.

Another prominent focus in the context of sports-cognitive relationships involves the distinctive performances of athletes in both domain-general and domain-specific cognitive processes. The broad transfer hypothesis posits that intensive practice in specific skills can bolster cognitive capabilities beyond the context of sport-specific scenarios, thus suggesting a spillover effect of enhanced cognitive abilities into broader domains [37]. A meta-analysis conducted by Kalén et al. [38] revealed parallel expert advantages in both domain-general and domain-specific higher-level cognitive functions, thus highlighting the potential for broad cognitive enhancements resulting from specialized training. Nonetheless, in the context of table tennis, a disparity has been identified: experts exhibit a more pronounced advantage in response inhibition with regard to tasks that are relevant to their sport than in tasks that are viewed as irrelevant [39,40]. This finding raises questions regarding the existence and extent of domain-related benefits in cognitive conflict control. Given that spatial information plays a more integral role in table tennis than does color information—i.e., athletes must continuously adapt their responses based on the ball’s spatial trajectory, including its spin type—a visuospatial judgment is viewed as a core competency that critically influences competition outcomes [41]. Furthermore, prolonged engagement in table tennis has been linked to enhanced visuospatial working memory [42], and studies that have utilized a spatial-cued Flanker task, such as that conducted by Wang et al. [43], have demonstrated that table tennis athletes exhibit superior executive control. In light of the fact that the connection between visuospatial abilities and table tennis experience is more pronounced than the connection between color-related information and such experience, it is becoming increasingly important to investigate whether experts have specific expertise-related advantages in different cognitive tasks, namely, the color-word Stroop task and the spatial Stroop task.

In summary, no research has yet explored the behavioral and electrophysiological aspects of domain-general and domain-specific cognitive inhibition processes in the context of table tennis athletes. Our current study seeks to fill these research gaps by employing two experimental approaches complemented by ERP measurements. In Experiment 1, the color-word Stroop task was employed to evaluate domain-general cognitive inhibition capabilities. Subsequently, Experiment 2 used the spatial Stroop task to assess domain-specific cognitive inhibition, thus investigating the nuances of spatial processing in a manner relevant to table tennis expertise. Based on the literature, we hypothesized that athletes would outperform nonathletes in both tasks and exhibit reduced neural activation associated with cognitive inhibition. Moreover, we expected that expert athletes would exhibit pronounced advantages in spatial Stroop tasks as compared to color-word Stroop tasks due to the specialized demands of their sport and their adapted cognitive processing strengths.

## 2. Materials and Methods

### 2.1. Participants

This study was conducted between February to April 2023 at the center for Exercise and Brain Science of Shanghai University of Sport. Participants were recruited through online advertisements at Shanghai Sports University. Participants were anonymous and volunteered to be included in the study. In addition, none of the recruited participants refused to participate in the experiments. The participant inclusion criteria were as follows: individuals had to be native Chinese speakers, reported normal or corrected-to-normal vision and no psychological or neurological disorders. For the table tennis athletes, eligibility required intensive training in table tennis over the preceding three months and achievement of at least the second level as per the Chinese national standard. The nonathlete group consisted of participants who had no prior training in table tennis or any other racket sports [44]. To mitigate the impact of physical activity levels, the non-athlete group was specifically selected from individuals who engaged in regular exercise. Demographic information about the participants was gathered through direct questioning, and their physical activity levels were quantified using the International Physical Activity Questionnaire (IPAQ; [45]).

Using G*Power 3 [46] to calculate 95% power (α = 0.05) for two-way mixed-measures analyses of variance (two-way ANOVAs), and based on the mean effect size (0.34) for high-level cognitive functions from a recent meta-analysis [38], it was determined that a total of 32 participants would suffice for the study. To further enhance the statistical robustness, we ultimately recruited 40 participants, including 20 table tennis athletes (11 males and 9 females; mean age ± standard deviation = 20.75 ± 2.24 years) and 20 nonathletes (9 males and 11 females; mean age ± standard deviation: 19.80 ± 1.85 years). The table tennis experience among athletes varied from 7.64 to 10.23 years, with an average of 9.58 ± 1.4 years. The highest level of competition achieved by athletes was at the national level. The majority of nonathletes were first and second-year students majoring in Sports Human Science, Public Administration, and related fields.

In addition, a survey utilizing the IPAQ was conducted to assess the physical activity levels of participants. The IPAQ is a seven-item scale that assesses physical activity over the previous week and has demonstrated good validity and reliability, with an intraclass correlation coefficient of 0.704 [45]. We calculated the metabolic equivalent (MET) by multiplying the duration of weekly activity by 8 METs for vigorous activity, 4 METs for moderate activity, and 3.3 METs for walking [47]. The results showed no significant difference in METs/week between table tennis athletes (3017.58 ± 474.93) and nonathletes (2392.55 ± 303.86), with t _(38)_ = 1.11, *p* = 0.28, indicating comparable levels of physical activity between the two groups.

### 2.2. Apparatus

All experiments were designed and executed using MATLAB (R2019a, The MathWorks, Natick, MA, USA) alongside PsychToolbox [48]. The stimuli were displayed on a 30-inch monitor, boasting a resolution of 1980 × 1470 pixels and a refresh rate of 60 Hz. The viewing distance was maintained at approximately 70 cm from the screen for participants. For the collection of electrophysiological data, a 64-channel electroencephalographic (EEG) system was employed, recording at a sampling rate of 500 Hz using the actiChamp system (Brain Products Inc., Gilching, Germany).

### 2.3. Stimuli and Procedures

In Experiment 1, the stimuli consisted of Chinese words representing RED (红) and BLUE (蓝), which were displayed in red (the color model of red, green, and blue; RGB: 255, 0, 0) and blue (RGB: 0, 0, 255), respectively. For Experiment 2, the stimuli comprised Chinese words representing UP (上) and DOWN (下), both displayed in black (RGB: 0, 0, 0). Across both experiments, two types of Stroop conditions (congruent and incongruent) were delineated based on whether the ink color (in Experiment 1) or the location (in Experiment 2) matched the semantic meaning of the words. The visual angle for each stimulus was set at 1.2° × 1.2°.

**Figure 1:**
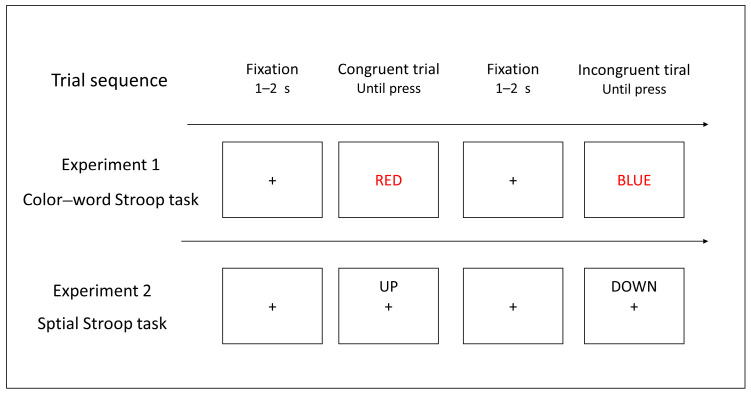
The procedures involved in the color-word Stroop task (Experiment 1) and the spatial Stroop task (Experiment 2).

As depicted in Figure 1, the procedure for both experiments commenced with a fixation cross displayed in the center of the screen for a duration of 1–2 s, setting the stage for the presentation of the target stimulus. In Experiment 1, the target stimuli were positioned at the center of the screen, while in Experiment 2, they appeared either above or below the central fixation point. Participants were tasked with making judgments regarding the targets both swiftly and accurately. Specifically, in Experiment 1, the challenge was to identify the color of the words presented, and in Experiment 2, the task shifted to determining the spatial location of the words. In each experiment, participants completed a practice block consisting of 10 trials, followed by a formal block containing a total of 120 trials, with an equal distribution across each condition. Participants completed both experiments with a counterbalanced order. The duration of each experimental session lasted approximately 10 min.

### 2.4. EEG Data Recording and Preprocessing

Two experiments recorded EEG data. Impedances were maintained below 5 kΩ, and the participants were instructed to avoid eye movements, blinking, and body movements as much as possible. EEG data preprocessing was conducted using the EEGlab toolbox (version 2023.0) in MATLAB (version R2023a). The data were re-referenced to the average of all electrodes and filtered with a low-pass frequency of 25 Hz and a high-pass frequency of 0.1 Hz. Data periods with large movement-related artifacts were removed using independent component analysis. Bad channels were replaced with data interpolated from good channels. Epochs were then segmented from −200 to 1000 ms. All epochs were baseline-adjusted using a 200 ms pre-stimulus period. Epochs with amplitudes exceeding ±100 μV were excluded from the analysis. Trials in which there were response errors were also excluded. After exclusions, the average for each condition per participant comprised at least 47 epochs. Separate grand-average waveforms were constructed across all participants according to the stimulus types.

Based on previous Stroop research, we quantified the N_inc_ and LSP components. The N_inc_ component was defined as the negative amplitude within 200–500 ms after stimulus onset on frontal electrodes [29]. The LSP component was defined as the positive amplitude within 400–1000 ms on the parietal electrodes [49]. According to the grand-average waveforms and topographical mapping of our data, we calculated the mean N_inc_ amplitudes during the time windows of 300–400 ms for Experiment 1 and Experiment 2 in the regions of interest (ROIs: F1, Fz, F2). Additionally, mean LSP amplitudes were calculated during the time window of 500–650 ms for Experiment 1 and 350–500 ms for Experiment 2 in the ROIs (P1, Pz, P2).

### 2.5. Statistical Analysis

For each experiment, two-way ANOVAs were employed to evaluate the effects of group (athletes vs. nonathletes) × Stroop type (incongruent vs. congruent) on ACC, RTs, N_inc_ and LSP amplitudes. In cases where a significant interaction emerged, additional independent *t*-tests were conducted to compare Stroop effects between groups. Additionally, correlation analysis was conducted to estimate the relationship between RTs and the amplitudes of N_inc_ and LSP components. Only RTs from correctly answered trials were included in the analysis, and any RTs that deviated by more than three standard deviations from the mean were excluded to eliminate outliers.

All the statistical analyses were conducted in MATLAB (version R2023a). A two-tailed alpha level of 0.05 was set as the significance threshold, and probability values were adjusted when appropriate using Greenhouse–Geisser epsilon correction for non-sphericity. We computed partial eta-squared ($\eta_{p}^{2}$) to determine effect sizes for both the main and interaction effects [50]. In cases where significant main effects were detected, we conducted a Bonferroni post hoc test to pinpoint specific differences. The mean and standard error (M ± SE) are provided for statistically significant results. Bayesian factors (BFs) were calculated for the ANOVA and t-test results to quantify the ratio between alternative and null hypotheses. BF_10_ can be interpreted as follows: null evidence, BF_10_ ≤ 1/3; inconclusive evidence, 1/3 < BF_10_ ≤ 3; moderate evidence, 3 < BF_10_ ≤ 10; strong evidence, 10 < BF_10_ ≤ 30; very strong evidence, 30 < BF_10_ ≤ 100; or extremely strong evidence, BF_10_ > 100, supporting the alternative, rather than null, hypothesis [51].

## 3. Results

### 3.1. Results of the Color-Word Stroop Task

The analysis of ACC revealed extremely strong evidence to support a significant main effect of Stroop type (F _(1, 38)_ = 28.71, *p* < 0.001, $\eta_{p}^{2}$ = 0.43, BF_10_ = 1.06 × 10^5^). This strongly suggested that participants tended to be more accurate in trials where the color and word were congruent (i.e., the word ‘red’ printed in red color) compared to trials where they were incongruent (i.e., the word ‘red’ printed in blue color), with an ACC difference of 3.76% ± 0.70%. Additionally, no significant group difference or group × Stroop type interaction on ACC was observed. This finding underscored the cognitive challenge posed by incongruent stimuli, which require more effort to process and respond to correctly. The analysis of RT revealed extremely strong evidence to support a significant Stroop effect (F _(1, 38)_ = 33.31, *p* < 0.001, $\eta_{p}^{2}$ = 0.47, BF_10_ = 1.74 × 10^4^). Participants responded faster in congruent conditions compared to incongruent conditions, with a RT difference of 34.20 ± 5.93 ms. This speed difference highlighted how congruency facilitates quicker cognitive processing and response generation. However, no significant group difference or group × Stroop type interaction on RTs was observed.

The N_inc_ amplitude analyses revealed inconclusive evidence to support a significant group difference (F _(1, 38)_ = 4.75, *p* = 0.036, $\eta_{p}^{2}$ = 0.11, BF_10_ = 2.07). Specifically, nonathletes exhibited more negative N_inc_ amplitudes compared to athletes, by an average difference of 1.75 ± 0.80 μV. This indicated that, from a neurological perspective, nonathletes might devote more cognitive resources to the detection of cognitive conflict than athletes. In addition, inconclusive evidence was found to support a significant Stroop effect (F _(1, 38)_ = 6.03, *p* = 0.019, $\eta_{p}^{2}$ = 0.14, BF_10_ = 2.07). Post hoc comparisons revealed that incongruent stimuli elicited more negative N_inc_ amplitudes than did congruent stimuli (difference = 0.44 ± 0.19 μV). This suggested that incongruent stimuli might require more neural effort to process, reflecting the cognitive challenge they pose. No group × Stroop type interaction on N_inc_ was observed. Analysis of the LSP amplitudes revealed strong evidence to support a significant Stroop effect (F _(1, 38)_ = 10.47, *p* = 0.003, $\eta_{p}^{2}$ = 0.22, BF_10_ = 11.96). The incongruent stimuli elicited more positive LSP amplitudes than did the congruent stimuli (difference = 0.47 ± 0.14 μV). In addition, inconclusive evidence was found to support a significant group × Stroop type interaction on LSP (F _(1, 38)_ = 5.20, *p* = 0.028, $\eta_{p}^{2}$ = 0.12, BF_10_ = 1.77). Post hoc comparisons revealed that the Stroop effect on LSP amplitudes was significant among nonathletes (0.80 ± 0.20 μV, *p* < 0.001) and stronger than among athletes (0.14 ± 0.20 μV, *p* = 0.503; t _(38)_ = 2.28, *p* = 0.028, BF_10_ = 1.33). This suggests that nonathletes might experience a more intense cognitive processing challenge when faced with incongruent stimuli than their athlete counterparts. No main effect of group on LSP amplitudes was observed.

**Figure 2:**
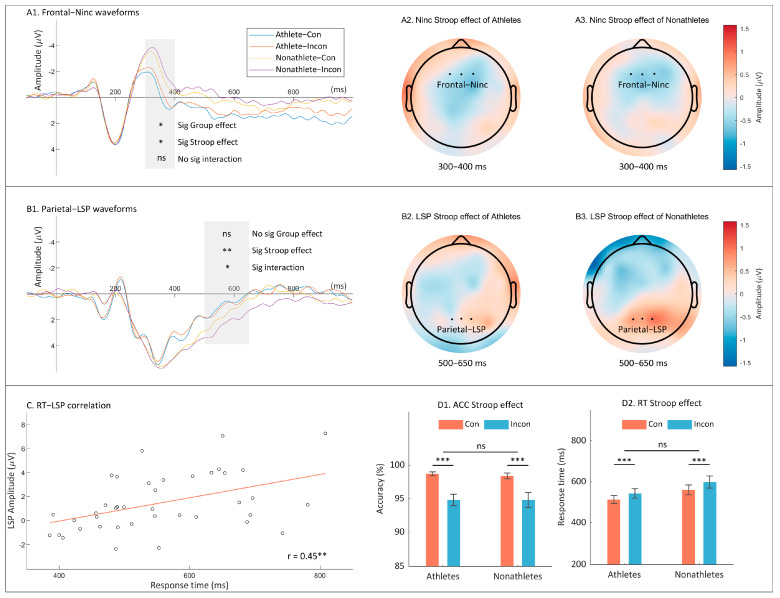
A comprehensive overview of both behavioral and electrophysiological findings pertaining to the color-word Stroop task. The following results are shown: the grand average waveforms of the N_inc_ amplitudes at the frontal electrodes (**A1**); the topographic scalp distribution of Stroop effect on N_inc_ amplitudes among both athletes (**A2**) and nonathletes (**A3**); the grand average waveforms of the LSP amplitudes at the parietal electrodes (**B1**); the topographic scalp distribution of LSP amplitudes of Stroop effect on among both athletes (**B2**) and nonathletes (**B3**); the correlation between RTs and LSP amplitudes (**C**); and the means and standard errors for each condition on ACC (**D1**) and RT (**D2**). In subplot C, each dot represents the data points correlating RT and LSP amplitude for each participant, the red line depicts the trend of this correlation. The term “(In)Con” denotes the (in)congruent condition, while “sig” indicates the significance of each effect. “ns” *p* > 0.05, *, *p* < 0.05, ** *p* < 0.01, *** *p* < 0.001.

Furthermore, the correlation analysis revealed a significant positive relationship between RTs and LSP amplitudes (r = 0.45, *p* = 0.003), meaning that LSP amplitudes increased alongside RTs. This relationship underscored the link between cognitive processing effort, as reflected in LSP amplitudes, and the time taken to resolve cognitive conflicts, as seen in RTs. However, no significant correlation was observed between RTs and N_inc_, highlighting the specific role of LSP amplitudes in reflecting cognitive effort in this context. Figure 2 displays the behavioral outcomes, ERP waveforms, topographic scalp distributions and the correlations between RTs and LSP amplitudes in Experiment 1.

### 3.2. Results of the Spatial Stroop Task

Two-way ANOVA of ACC found moderate evidence to support a significant Stroop effect (F _(1, 38)_ = 7.71, *p* = 0.009, $\eta_{p}^{2}$ = 0.17, BF_10_ = 7.34), ACC in the congruent condition was higher than that in the incongruent condition (difference = 1.08% ± 0.39%). No significant group main effect and group × Stroop type interaction on ACC was observed. The analysis of RT revealed moderate evidence to support a main group effect (F _(1, 38)_ = 9.36, *p* = 0.004, $\eta_{p}^{2}$ = 0.20, BF_10_ = 9.23). Athletes respond faster than nonathletes (difference = 58.35 ± 19.02 ms). In addition, moderate evidence was found to support a significant Stroop effect (F _(1, 38)_ = 9.36, *p* = 0.004, $\eta_{p}^{2}$ = 0.20, BF_10_ = 8.00). The RTs in the congruent condition were faster than those in the incongruent condition (difference = 10.15 ± 3.32 ms). Finally, we found strong evidence supporting a significant group × Stroop type interaction on RTs (F _(1, 38)_ = 10.79, *p* = 0.002, $\eta_{p}^{2}$ = 0.22, BF_10_ = 12.89). Simple effect analysis found that the Stroop effect of nonathletes (21.05 ± 4.69 ms, *p* < 0.001) was significant and larger than that of athletes (0.01 ± 4.69 ms, *p* = 0.87; t _(38)_ = 3.28, *p* = 0.002, BF_10_ = 6.94).

**Figure 3:**
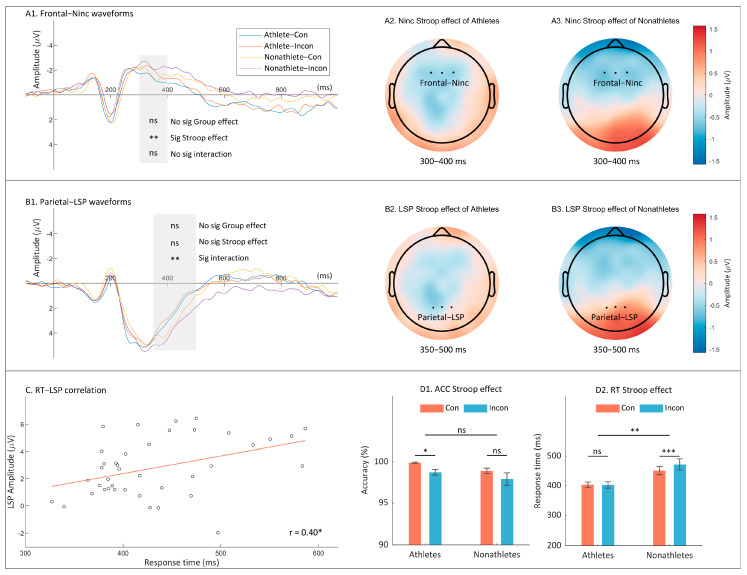
A comprehensive overview of both behavioral and electrophysiological findings pertaining to the spatial Stroop task. The following results are shown: the grand average waveforms of the N_inc_ amplitudes at the frontal electrodes (**A1**); the topographic scalp distribution of Stroop effect on N_inc_ amplitudes among both athletes (**A2**) and nonathletes (**A3**); the grand average waveforms of the LSP amplitudes at the parietal electrodes (**B1**); the topographic scalp distribution of Stroop effect on LSP amplitudes among both athletes (**B2**) and nonathletes (**B3**); the correlation between RTs and LSP amplitudes (**C**); and the means and standard errors for each condition on ACC (**D1**) and RT (**D2**). In subplot C, each dot represents the data points correlating RT and LSP amplitude for each participant, the red line depicts the trend of this correlation. The term “(In)Con” denotes the (in)congruent condition, while “sig” indicates the significance of each effect. “ns” *p* > 0.05, *, *p* < 0.05, ** *p* < 0.01, *** *p* < 0.001.

The N_inc_ amplitude analyses revealed moderate evidence to support a significant Stroop effect (F _(1, 38)_ = 8.56, *p* = 0.006, $\eta_{p}^{2}$ = 0.18, BF_10_ = 6.21). Post hoc comparison found that incongruent stimuli triggered more negative N_inc_ amplitudes than congruent stimuli (difference = 0.57 ± 0.19 μV). No significant group main effect or group × Stroop type interaction on N_inc_ was observed. The LSP amplitude analyses revealed moderate evidence supporting a significant group × Stroop type interaction (F _(1, 38)_ = 9.15, *p* = 0.004, $\eta_{p}^{2}$ = 0.19, BF_10_ = 9.21). Simple effect analysis found that the Stroop effect on LSP amplitudes was significant among nonathletes (0.56 ± 0.19 μV, *p* = 0.006) and stronger than among athletes (−0.27 ± 0.19 μV, *p* = 0.17; t _(38)_ = 3.03, *p* = 0.004, BF_10_ = 9.30). Furthermore, nonathletes showed more positive LSP amplitudes than athletes in incongruent trials (difference = 1.54 ± 0.67 μV, *p* = 0.03) rather than those in congruent trials (difference = 0.71 ± 0.68 μV, *p* = 0.30). No main effect of group on LSP amplitudes was observed. Furthermore, the correlation analysis revealed a significant positive relationship between RTs and LSP amplitudes (r = 0.40, *p* = 0.010). LSP amplitudes increased alongside RTs. No significant correlation was observed between RTs and N_inc_. Figure 3 displays the behavioral outcomes, ERP waveforms, topographic scalp distributions and the correlations between RTs and LSP amplitudes in Experiment 2.

## 4. Discussion

To our knowledge, this study represents the first exploration of cognitive inhibition abilities and associated neural activities in the context of table tennis expertise. We assessed the performance of table tennis athletes and nonathletes across color-word and spatial Stroop tasks using enhanced ERP analyses. The results confirmed our hypotheses, thus demonstrating that table tennis athletes outperformed nonathletes in both tasks and exhibited particularly significant advantages in the spatial Stroop task. These findings lend credence to the neural efficiency hypothesis and deepen our understanding of the relationship between cognitive functions and table tennis expertise.

In the color-word Stroop task, evidence indicated that athletes exhibit benefits from more efficient neural processing. We discovered that athletes did not outperform nonathletes behaviorally, a finding which was consistent with previous Stroop research findings on athletes in other domains [52,53]. Nevertheless, discrepancies in the ERP components between the groups were detected. Compared with nonathletes, athletes displayed reduced N_inc_ amplitudes and smaller Stroop effects on LSP amplitudes. According to prior research, the N_inc_ component is indicative of an early conflict detection subprocess in the context of cognitive inhibition, whereas LSP pertains to the conflict resolution phase [28]. Notably, in trials featuring correct responses, diminished conflict-related amplitudes have been associated with more effective inhibitory control [31]. Thus, these findings suggest that athletes utilized fewer cognitive resources to detect and resolve color-word conflicts than nonathletes did, despite the fact that their behavioral outcomes were comparable. This observation is supported by the study by Li et al. [54], in which context elderly individuals who regularly engaged in table tennis exhibited diminished N2 amplitudes in color-word Stroop tasks as compared to irregular exercisers. However, the accuracy benefits of table tennis athletes observed in previous research with Golden’s Stroop tests [35,36] were not replicated in our research. This suggested different cognitive models between “reading” and “laboratory-based perceptual-motor” responses. Additionally, other studies have shown that athletes in sports such as basketball [52] and martial arts [53] do not outperform nonathletes in the color-word Stroop test, challenging the idea that athletes have a general cognitive inhibition advantage. Despite these findings, our results indicate that a thorough examination of both neural activity and behavioral performance is essential to accurately assess the presence of an expert advantage.

In the spatial Stroop task, table tennis athletes benefited from both efficient neural processing and behavioral performance. The data provided moderate to strong evidence indicating that athletes respond more quickly and exhibit reduced Stroop effects on RTs and LSP amplitudes as compared to nonathletes. Additionally, the positive correlation observed between RTs and LSP amplitudes suggested that the reaction is associated with late selective motor processes. Consistent with [22], athletes exhibited superior behavioral performance despite exerting less brain control during conflict resolution processes. The observation regarding decreased brain control among athletes in both Stroop tasks lends support to the neural efficiency hypothesis [24], thus suggesting that long-term training may cause brain plasticity to enhance inhibitory efficiency [23,55]. Previous resting-state comparisons between table tennis athletes and nonathletes have revealed that athletes exhibit improved structural integrity with regard to white matter fiber bundles [56] and superior brain functional connections [57]. These training-induced neural adaptations, which are indicative of efficient connectivity, have been identified in athletes who participate in racket sports, including tennis and table tennis [58]. Furthermore, a correlation between lower activation in the frontoparietal attention network and higher world rankings among table tennis players has been documented, thus reinforcing the claim that specific neural efficiencies contribute significantly to elite sports performance [59]. This body of evidence collectively highlights the ability of specialized, long-term table tennis training to foster cognitive and neural advantages.

Significantly, we observed unique patterns of cognitive inhibition related to table tennis experience in both Stroop tasks. Group differences were characterized by the initial activation of the frontal lobes and the subsequent involvement of the parietal lobes in the process of managing color-word conflict inhibition. In contrast, the late-stage conflict control exerted by the parietal lobes, alongside behavioral benefits, was evident during the inhibition of spatial conflicts. In light of the intrinsic characteristics of table tennis, which requires quick spatial judgments and reactions [19,41], the spatial Stroop task more accurately mirrors the cognitive demands that athletes face in their sport than does the color-word Stroop task. Therefore, on one hand, our insights into brain characteristics provide support for the notion of expertise transfer from sport-related domains to broader cognitive realms [60]. On the other hand, the behavioral advantages that were identified exclusively in the context of spatial Stroop tasks suggest that table tennis athletes exhibit heightened cognitive benefits in domain-specific tasks, a finding which is consistent with earlier studies on response inhibition [20,22,39,40]. Moreover, cognitive inhibition underscores the role of selective attention [61,62], which utilizes top-down control to focus on relevant information. Accordingly, the distinct models of expert advantages observed in our two Stroop tasks suggest that athletes’ control over attention varies based on domain relevance. This variation highlights how athletes adapt their cognitive strategies to align with the specific demands of different tasks, showcasing a sophisticated level of attention modulation influenced by their training and expertise.

The MGI model, as described in detail by Ullén et al. [7], highlights the crucial role of deliberate practice and its interaction with working memory, which can enhance the domain-specific advantages of experts. More specifically, this model suggests that through deliberate practice, sports-related information is encoded into long-term memory, thus facilitating its efficient retrieval during domain-specific tasks. When this retrieval process is combined with superior executive control, it can significantly enhance athletes’ performance, potentially reflecting their innate cognitive capabilities, as discussed by Simonet et al. [63]. Moreover, some scholars have argued that the primary cognitive benefits that athletes exhibit are closely connected to the specific sports in which they engage [8,64]. Overall, these insights not only elucidate the nuanced relationships between specific types of cognitive inhibition and table tennis expertise but also highlight the potential for sports training to promote targeted cognitive improvements.

Although our study provides valuable insights, it also has several limitations. First, the BF_10_ values observed in the context of the color-word Stroop task yielded inconclusive evidence regarding the positive differences in ERPs between groups. This outcome highlights the need for further research to identify the scope of table tennis athletes’ neural processing advantages in such tasks more precisely. Second, our exploration of particular correlations relied heavily on the relevance of color and spatial attributes to table tennis. Previous research in the field of table tennis has often utilized materials related to sports scenarios to establish domain relevance [38,65], acknowledging that these scenarios contain complexities that far surpass those represented in our spatial Stroop task. Nonetheless, our findings suggest that altering only one dimension of a stimulus can significantly affect expert performance, thus highlighting the need for more sophisticated experimental designs to be employed in future studies to improve our understanding the domain-specific cognitive advantages of athletes. Third, although the importance of training experience to expert advantages has been emphasized, employing the methodological approach of cross-sectional comparison in isolation is insufficient to establish causality. Additionally, the MGI model highlights the necessity of considering a wider array of factors beyond the level of mere practice. Elements such as personality, interests, empathy and motivation play critical roles in shaping expertise [7] and influencing cognitive function [66]. Integrating these elements into the framework is necessary to obtain a holistic understanding of how various factors contribute to the development of expert skills [67]. Fourth, given the small sample size in our study, the results may be subject to selection bias [38] and individual variability [68]. Future research should consider expanding the sample size and accounting for individual differences to strengthen the robustness of the conclusions. Finally, while ERP techniques offer a high degree of temporal resolution, they also exhibit low spatial resolution. Future studies could explore the brain mechanisms underlying the effects of table tennis expertise on cognitive inhibition in further depth, such as by utilizing fMRI or MEG measurements to obtain enhanced spatial insights. Although our study is limited by its cross-sectional design and the inherent constraints of ERP techniques, it lays the groundwork for future research to explore these cognitive processes by employing more sophisticated experimental designs and methodologies.

## 5. Conclusions

In conclusion, our study contributes to the growing body of literature on the cognitive benefits of specialized training in sports, specifically in the context of table tennis. By employing both the color-word and spatial Stroop tasks alongside ERP measurements, we have reported evidence indicating that table tennis athletes exhibit enhanced cognitive inhibition abilities, particularly in tasks that closely mirror the spatial demands of their sport. Specifically, in the color-word Stroop tasks, athletes demonstrated advantages through more efficient neural processing, as evidenced by decreased N_inc_ (300–400 ms) amplitudes and a reduced Stroop effect on LSP (500–650 ms) amplitudes compared to nonathletes. In the spatial Stroop tasks, athletes benefited from both efficient neural processing and behavioral performance, as indicated by reduced Stroop effects on LSP (350–500 ms) and RTs. These findings support the neural efficiency hypothesis and deepen our understanding of how cognitive abilities interact with table tennis expertise. Building on these insights, the practical implications of our findings could be applied to developing targeted training programs that enhance cognitive skills in athletes, potentially improving performance in competitive scenarios. Additionally, future research should explore these cognitive processes further by employing longitudinal studies to track changes over time and by integrating more nuanced measures of cognitive function to confirm and expand upon our findings.

## Data Availability

The data set supporting the conclusions of this article will be made available by the authors on reasonable request. The data are not publicly available due to ongoing analysis and additional research projects that are building upon this dataset.
